# The ethical challenges of personalized digital health

**DOI:** 10.3389/fmed.2023.1123863

**Published:** 2023-06-19

**Authors:** Els Maeckelberghe, Kinga Zdunek, Sara Marceglia, Bobbie Farsides, Michael Rigby

**Affiliations:** ^1^Beatrix Children’s Hospital, University Medical Center Groningen, University of Groningen, Groningen, Netherlands; ^2^Health Education Unit, Medical University of Lublin, Lublin, Poland; ^3^Faculty of Clinical Engineering, University of Trieste, Trieste, Italy; ^4^Brighton and Sussex Medical School, University of Sussex, Brighton, United Kingdom; ^5^School of Social, Political and Global Studies and School of Primary, Community and Social Care, Keele University, Keele, United Kingdom

**Keywords:** ethics, personalized health, trust, consent, connectivity, evidence, evaluation, policy

## Abstract

Personalized digital health systems (pHealth) bring together in sharp juxtaposition very different yet hopefully complementary moral principles in the shared objectives of optimizing health care and the health status of individual citizens while maximizing the application of robust clinical evidence through harnessing powerful and often complex modern data-handling technologies. Principles brought together include respecting the confidentiality of the patient–clinician relationship, the need for controlled information sharing in teamwork and shared care, benefitting from healthcare knowledge obtained from real-world population-level outcomes, and the recognition of different cultures and care settings. This paper outlines the clinical process as enhanced through digital health, reports on the examination of the new issues raised by the computerization of health data, outlines initiatives and policies to balance the harnessing of innovation with control of adverse effects, and emphasizes the importance of the context of use and citizen and user acceptance. The importance of addressing ethical issues throughout the life cycle of design, provision, and use of a pHealth system is explained, and a variety of situation-relevant frameworks are presented to enable a philosophy of responsible innovation, matching the best use of enabling technology with the creation of a culture and context of trustworthiness.

## 1. Introduction – long-standing and new challenges accentuated by digitized personal health

### 1.1. Transformative methods bring related challenges

The personalization of healthcare is a foundational principle from the earliest code of medical ethics. Now, after two centuries of primarily static paper-based recording and communication methods, electronic digitized technologies for data capture, processing, and communication open radical new opportunities to enable the personalization of care and the harmonization of contributing professional components, but this unprecedented opportunity brings concurrent new challenges in the need to blend foundational principles with the maximization of new benefits.

Digitization in healthcare is an aggregation of the newest technologies throwing up its own challenges within data management and healthcare delivery. Ongoing organizational, methodological, and technological advancements lead to a complex transformation of health and social care toward personalized, participative, preventive, predictive, and precision medicine (characterized as 5P medicine) (1). Such 5P medicine ecosystems are highly complex, dynamic, multidisciplinary, context-sensitive, knowledge-driven, and policy-controlled. The ecosystem is structurally and functionally characterized by its components representing specific aspects of the system, their functions, and relationships as well as the interaction of the system with its environment. The challenge is to define all impacting domains and to formally represent the related knowledge for the concrete use case of each specific business system. To ensure quality, consistency, and trust, the ISO 23903:2021 interoperability and integration reference architecture – model and framework (2) should be used. Policies that should control the behavior of 5P medicine business systems during their design and deployment include legal constraints, procedural requirements, and security and privacy concerns, all being focused on the goal of individual expectations and wishes of patients and the related practice decisions of professionals. All these should be underpinned and guided by ethical principles. The paper will address the ethical domain in this holistic context.

### 1.2. Personal yet informed – two key healthcare dichotomies spanning the millennia

The mission of pHealth brings together across three millennia old and new challenges of trust, risk, and ethics related to healthcare delivery. The key to the success of pHealth is the effective use of medical and personal data, which is an ethically sensitive issue even at the simplest level, and particularly so when using technological tools which in their details (and controls) are unfamiliar to many of those served. It juxtaposes four challenging concepts – the use of information embedded in the confidential patient-clinician dialogue; the use of extended personal case history to give a full longitudinal picture; sharing with co-creators of care to enable smooth holistic service delivery; and, the utilization of composite medical knowledge distilled from population-level personal health outcomes.

The Hippocratic Oath of *circa* 400 BC is firmly grounded on the health professional’s interaction with the individual regardless of their status, but at the same time also emphasizes the practitioner’s dependence on their teacher (and thus on antecedent knowledge) and on the importance of deferring to the superior technical skills of others (3). Two millennia later, in 1623, Donne emphasized that “No man is an island … because I am involved in mankind…” (using “man” in the historic representation of “person”) (4). Nowhere is this more true than in medicine and healthcare where medical knowledge can only be built up by the creation of insight from the epidemiology and treatment outcomes of a wide population – but the delivery of that care should be personalized and confidential, yet it is often shared within a virtual team.

For optimal and ethical health care delivery, the health professional should not work outside personal knowledge (and related locus of practice) but at the same time should competently access and utilize the full body of relevant health evidence in the specific illness or practice field. Moreover, this knowledge should be applied in a way moderated to match the personal presentation and characteristics of the subject of care. From this arises an essential need, identified from Hippocrates onward, to exploit cumulative health knowledge and relate it through the treating clinician back to the situation of the presenting individual – a daunting task unless well supported by the methods and resources of the day.

### 1.3. The computer as a powerful enabler

Computing – the rapid processing of standardized data items and presentation of calculated results – brought a new and powerful tool to address this challenge. In the mid-20th century, the power of computers was starting to be developed for healthcare purposes, and interest blossomed as the potential nature and scale of use became apparent.

In 1977, an influential paper considering the emerging opportunities and issues for informatics application in medicine focused on decision-making in medicine and opened with the following sentence (5):

*Medicine is a discipline of judgment and action. At each moment of his professional life, the physician must suggest decisions and actions to his patient. In order to do this, he must gather some pertinent information and extract in the most logical and the surest way arguments allowing him to achieve his objectives.*

**Figure 1:**
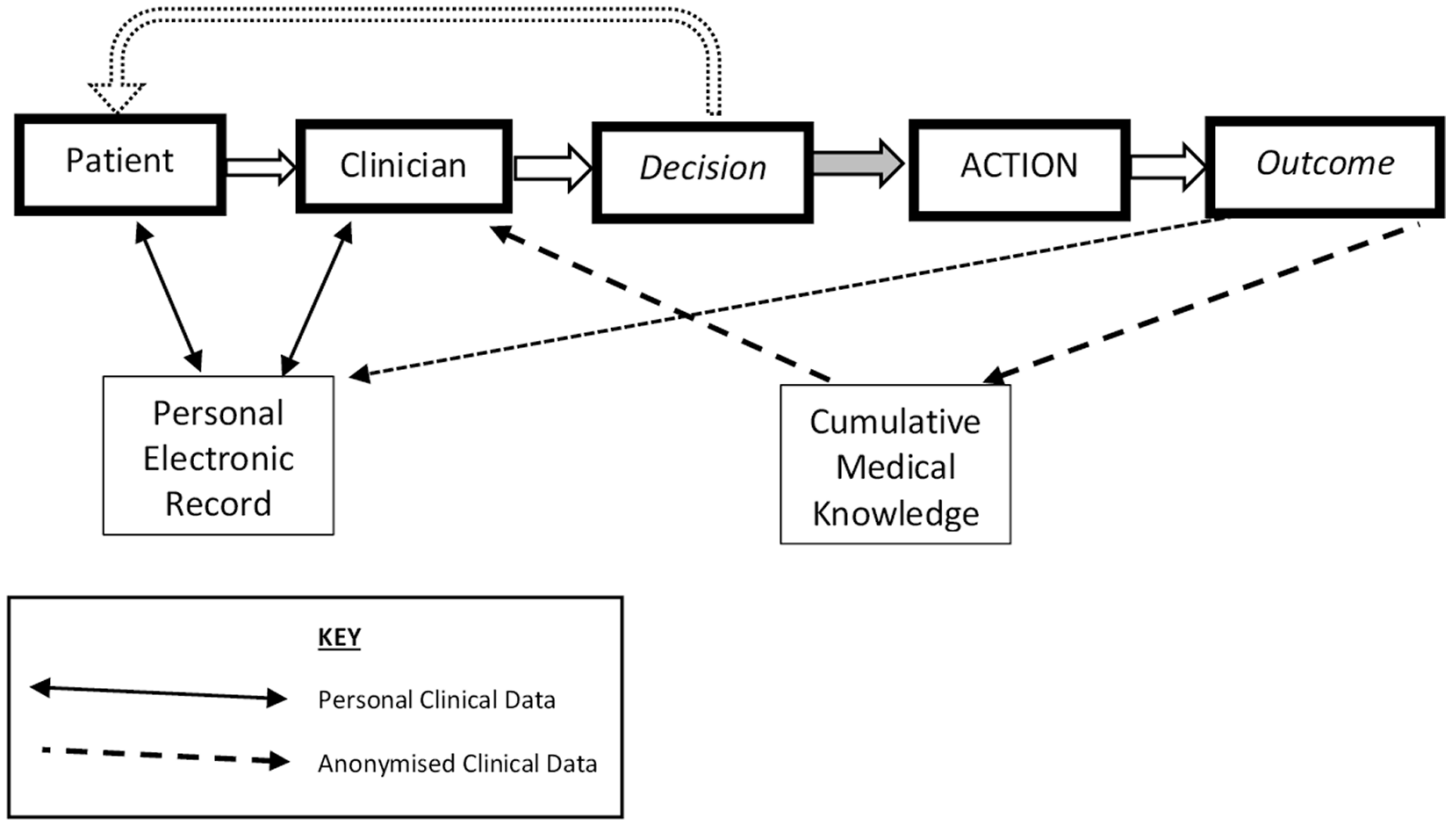
Types of personal data flow enabling decisions for evidenced Personalized Health. Developed from Grémy and Goldberg (5).

Apart from the archaic male personalization, this neatly sums up the computational task for clinical practice, and, thus, in modern terms, the safe delivery of personalized medicine. The paper mapped the healthcare decision processes and the related information dependencies, and this can be updated to the modern context of pHealth as shown in Figure 1, which indicates that it is the combination of the patient’s views and physical presentation and the knowledge gleaned from the electronic record and from emergent medical knowledge that the clinician analyzes in order to create a proposed course of action; this is then shared with the patient and the process is iterated as necessary.

The task for 21st-century pHealth is to accommodate the latest person-centric concepts and to ensure that all the components and the whole process meet ethical requirements. Within this, using computational support is very different from submitting to technological domination – the computer must be used responsibly and ethically as a controlled tool. As such, the art of medicine remains, with the clinician and the patient working together to engage responsibly designed technological tools in the interest of balancing competing interests, making fine judgments, and ensuring consistency of delivery.

### 1.4. Social responsibility and ethics in science and technology

Fortunately, the need to carefully define the role of technology within medical decision-making was recognized at a relatively early stage. Once the power of computers in health and healthcare became clear, commentators began to identify the need for responsibility and ethics. By 1992, Durbin published an article on Social Responsibility in Science, Technology, and Medicine (6). Subsequently, Beckwith and Huang wrote, “If society is to remain in step with new technology, the scientific community needs to be better educated about the social and ethical implications of its research” (7). Questions on Ethics, Computing and Medicine, and the informatics transformation of healthcare started to be explored (8). This issue has extended with the expansion of digital data gathering and communicating technologies to augment and extend the core computing function not least including the new opportunities but related bias and evidence risks of so-called Artificial Intelligence (AI) in health data analysis (9–11).

### 1.5. The expansion of ‘patient’ to ‘connected service user’

The sociological context of patienthood has been steadily developed, linking again to no ‘man’ being an island (4). No person should live in isolation, with the corollary that a person’s health condition is influenced by their immediate family and friends, as well as impacting them. In turn, those close contacts may be active in providing aspects of healthcare support; the ‘Patient’ in Figure 1 should be seen as a node interacting with formal and informal carers, necessitating authorized information flows, and finally, the patient and their close network should not be passive recipients of healthcare but should be involved in treatment and delivery decisions, allowing for preferences (12, 13). The World Health Organization has framed this in a Global Strategy on People-centered and Integrated Health Services (14). Thus, this special edition, and this paper, emphasizes the importance of “participative” alongside the other pHealth principles of the “5P” approach, namely, personalized, participative, preventive, predictive, and precision, while building on a recent framing of the ethical aspects (15, 16).

## 2. A holistic, design-based, and person-focused approach to ethics in pHealth

Based on an interdisciplinary architectural approach to pHealth (1) and the analysis of related trust aspects (17), this paper seeks to blend the ethical dimensions of healthcare principles and person-based values and expectations with new technology challenges. It also draws on the emerging understanding of Responsible Research and Innovation (RRI), which seeks to circumvent the false dichotomy between the ethics of research and the new ethical issues of changed roles, processes, and societal effects as innovation is rolled out (18–21). The claim is that the old and the new are mutually enabling if fundamental ethical principles are included as core design principles.

**Figure 2:**
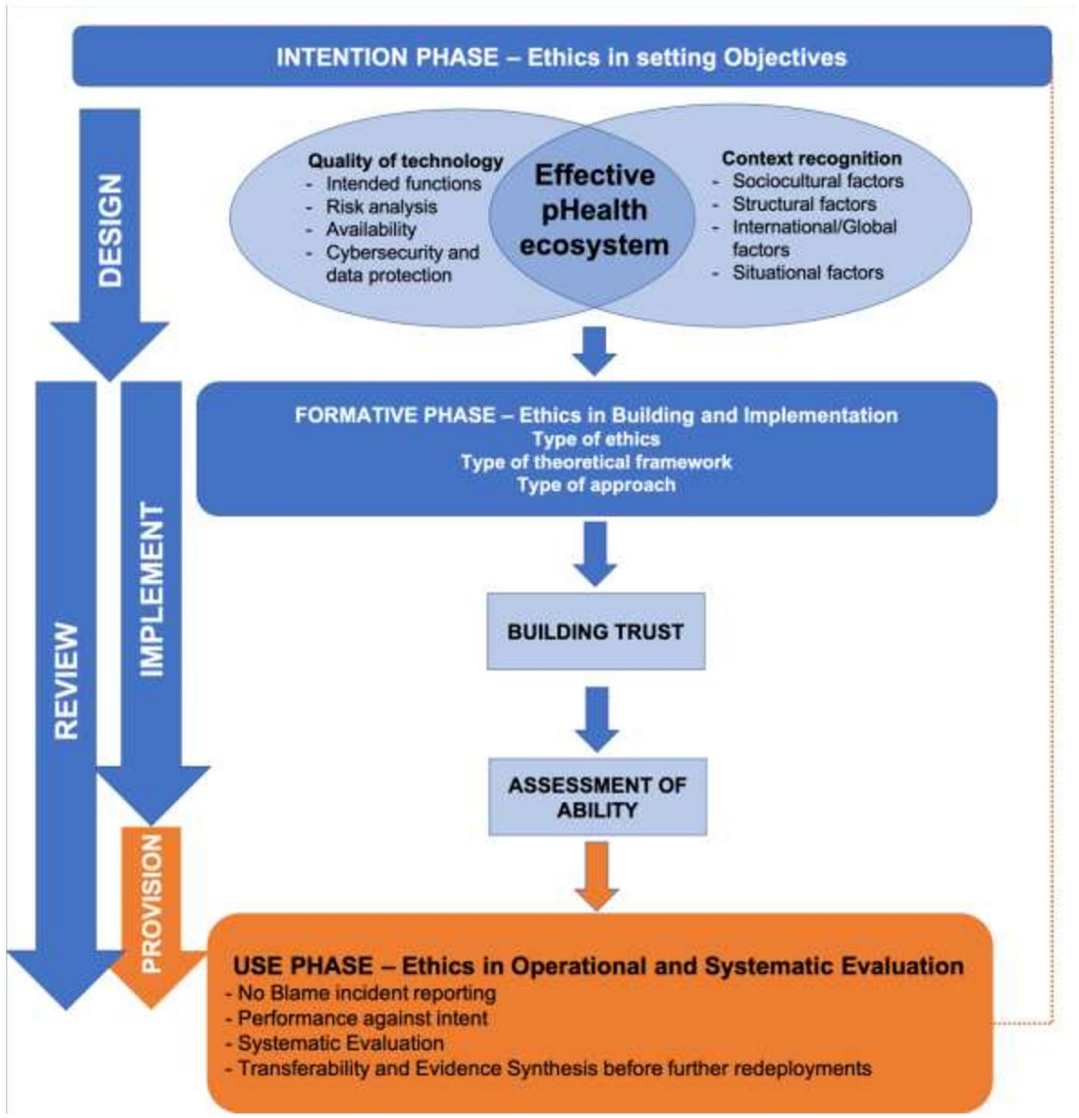
How Ethical Reflections should permeate pHealth Design and Application.

Moreover, it is both wrong and inefficient to treat ethical aspects as something to be applied retrospectively as a summative acceptance test – in order to be grounded and robust, and in order to avoid the need for post-build rectification, ethical principles need to be built into pHealth system development from the conception of objectives through to ensuring effective and equitable use in practice in line with Responsible Research and Innovation principles. Figure 2 shows how ethics need to be woven formatively through the life cycle of the endeavor.

Because pHealth embraces multiple contexts, it crosses several dimensions of ethics (15). These include the patient-practitioner interaction with medical ethics, the interaction of public health institutions embracing public health ethics, and the secondary use of data pertaining to the ethics of health research and the creation of accessible knowledge. Therefore, the ethical considerations on pHealth ecosystems could be required to navigate between and balance multiple considerations, and different frameworks and approaches have been described as Reflective Equilibrium (22), which has been shown to be applicable in modern health policy challenges (23).

This necessitates using a range of viewpoints and principles to initiate and support a normative discussion in order to reach a rational and defensible position as the issue progresses. Questions of technology interfacing with personalization are addressed in Section 3, while a review of the main principles, frameworks, and approaches to ethics which can be utilized is in Section 4.

### 2.1. Ethical approaches applied throughout pHealth design and build

The three aspects of applying ethics in the system process are:

#### 2.1.1. The intention phase (ethical objectives)

Whilst improved care delivery and clinical outcomes are often the initial trigger for pHealth and other health informatics initiatives, ethically, the core objectives when setting up any form of the health system should be equity of accessibility and acceptability. Inequity is often unintentionally built into health systems through hindrances which include practical access, differential health determinants, and restricted eligibility, and informatics systems can be postulated to ensure equity of advocacy (24). The utilization of data technology requires us to enquire as to how this tool might either enhance or impede our ethical intentions. For example, we might need to ask about the effect of any relevant digital divide in society, whereby many of those most in need of healthcare are less active with or do not trust digitally provided services (25).

Thus, the intention phase and the setting of objectives in a holistic and reflective mode provide a key opportunity to set any initial technical breakthrough or service efficiency objectives into a richer, balanced, and ethically underpinned holistic purpose. Included in this is moving away from concepts of ‘disadvantage’ and of ‘hard-to-reach’ patients toward equity of delivery and an acknowledgment of our past failures in underserving particular communities. User views and values should be incorporated, and any inherent difficulties or challenges should be addressed, and the risk of designing primarily for ‘people like us’ (26) and of building in new technological health inequities (27, 28) are, thereby, avoided. It is also important to manage expectations and avoid hyperbole from organizational or political sponsors with blinkered or institution-focused unrealistic aspirational goals.

#### 2.1.2. The formative phase (implementation ethics)

To make things happen at a practical level, there is a need to build and develop trust into a shared and enduring state of systemic trustworthiness (29, 30). This must apply across the infrastructures required to support data capture, record linkage, and maintain confidentiality and data security, as well as practices such as biobanking, data extraction storage, and interrogation. To build trust and trustworthiness, it is important not only to have a technically well-developed system but also to understand what matters to the people whose participation is crucial and to involve them from an early stage (12). In a wider engineering design context, the concept of Value-Sensitive Design has been developed with this in mind (31).

From the technical viewpoint, developers should ground trustworthiness on “quality” based on international evidence, guidelines, and recommendations. Quality is a process that runs throughout the development process from the early design phase. However, the quality and functioning of the technology are also dependent on effective adoption, and, again, a deliberative approach related to the system and context is needed to identify and apply ethical values (32). Since the adoption and effective use largely depend on context recognition, both the quality of technology and context recognition contribute to the design phase to implement an effective pHealth digital system (Figure 2).

#### 2.1.3. The use phase (provision of service ethics)

A strong moral claim would be that the only personalized medicine worth having is that which is genuinely available to every person based on an evidence base that reflects their lived experience and their biology and treats them equally according to need. This is in the context of the change in health care delivery model from ring-fenced individuals to connected citizens, with identified family members and informal carers. Ensuring that both professionals and patient and carer users have adequate levels of e-literacy (33) for their respective roles in the particular system context is a specific aspect of ensuring that implementation and use are ethical.

## 3. The intersection between technology and personalized health goals

Personalization is the action of designing a good or a benefit to meet someone’s individual requirements. When offering personalized care, the system needs to serve all citizens equally according to their needs. The ethical requirement is to meet these needs optimally and safely while allaying any anxieties and fears, optimizing the use of societal resources, and minimizing adverse effects on their familial and personal social context. This sets the framework for deliberation on the design and implementation approaches taken and sharing the rationale and justification. Herein lies the social responsibility of the doctor and other health professionals, and the requirement that patients acknowledge some sense of solidarity with others whose interests also need to be taken into account.

Personalization in a medical or health context is reflected in the concept of personalized medicine (PM) which seeks to make healthcare smarter and more efficient by integrating information from different sources. It is understood as “tailor-made prevention, diagnosis, and treatment for individuals or groups of individuals, enabling healthier and more productive lives” (34). Further, “The goal of personalized medicine is to optimize medical care and outcomes for each individual, resulting in an unprecedented customization of patient care” (35). PM is based on describing interventions that apart from the clinical patient profile, seek “biological information and biomarkers on the level of molecular disease pathways, genetics, proteomics, and metabolomics” (33, 36, 37), which is both promising and challenging.

Personalized medicine generally aspires that treatment will be directed to those patients who are more likely to receive benefits or not be harmed (37). Individually tailored therapies will result in higher possibilities in the field of disease prevention, improvement of survival rate, and extension of health span (38). However, “integration of personalized medicine into the clinical workflow requires overcoming several barriers in education, accessibility, regulation, and reimbursement” (35). Nevertheless, personalized medicine is a core principle of health optimization; it should mean that delivery is optimized to the needs of the patient and their immediate care team, thus maximizing uptake and effectiveness and minimizing carer disruption (12, 39).

### 3.1. Technological considerations

There are three element contexts for a pHealth service system:

- **Component** (such as input or output device, sensor, etc.),
- **Construct** of the components into a delivery system, and
- **Context** of service into which it is placed (treatment patterns, permitted reimbursed actions, etc.)

**Figure 3:**
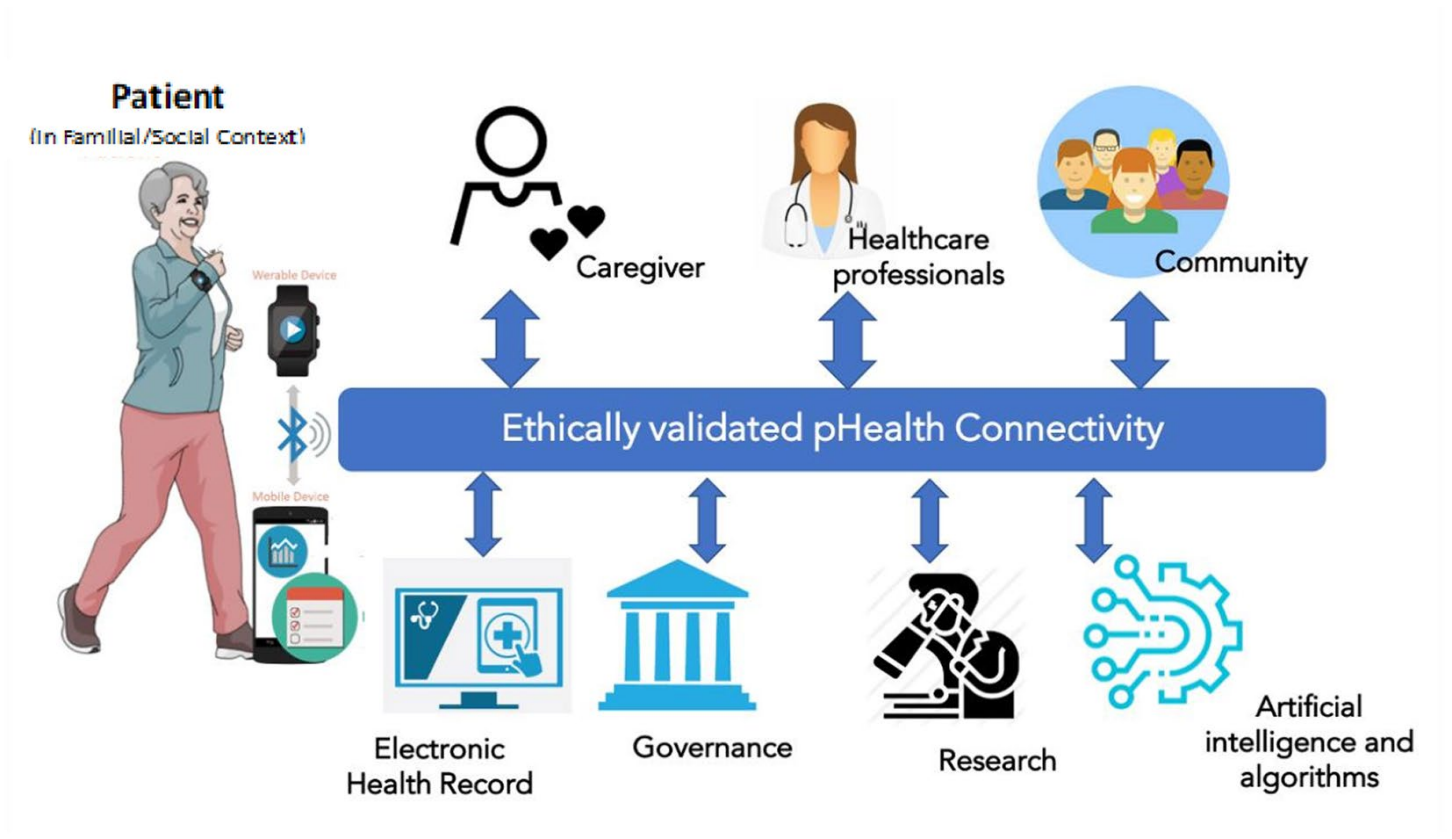
Actors, contexts, and technologies of a pHealth digital ecosystem.

pHealth digital systems should implement technologies able to serve the needs of personalized health, for instance, offering personalized services, collecting personal health information, informing patients with personalized content, and enhancing communication. This may lead to a simplistic view in which the pHealth digital system is at the intersection of mHealth (mobile applications for personalized services), Internet of Health Things (IoHT, for data collection and/or therapy delivery), and telemedicine (to enhance communication). However, while personalization refers to a single ‘patient’, the real-world application of personalized interventions creates a layer of complexity, as described in Figure 3.

The individual patient is always the starting point of the process, and mHealth apps, IoHT devices, and other software tools are used to collect personal health-related or environment-related data (activity, diet, habits, geo-localization, etc.) and support the patient in managing their health status (e.g., drug alerts, warnings, physical activity alerts, diet suggestions, etc.) This represents the overall “Virtual PHI repository” (17), in which personal data are not limited to those generated when the individual is a “patient” but also when she/he is still healthy. Clinical data in the electronic health record (EHR) are part of personal data managed within healthcare information systems. However, the defined challenges cannot be managed simplistically at the data level but have to consider the real-world business system, actors’ perspectives, contexts, experiences, skills, methodologies, languages, etc. (Figure 3), so the formalization and representation of the concepts and knowledge as described in the introductory paper (1) as well as in ISO 23903 (2, 40) is required.

Telecommunication and connectivity allow the subject to connect to all the relevant actors in the healthcare pathway, including caregivers, families, healthcare professionals, and reference communities such as patient associations. The same connectivity can be used to share data in a bi-directional fashion from the EHR to and from the patient. However, there are other actors in the picture. Data collected can have several “secondary” uses spanning from governance decision-making (e.g., drug surveillance and diagnostic appropriateness), to research, and also to the use of anonymous data in large, big data ecosystems for the possible use of artificial intelligence, cognitive computing, and machine learning. In the long run, the data generated and collected within a pHealth system will be relevant not only at the personal but also at the population level, in the light of “precision public health” (1). This complexity allows the transition from the simple definition of a pHealth digital system to a pHealth “ecosystem” (17), in which the common goal of all stakeholders is the patient. Solving this challenge requires the ontological representation of the business system and its domains involved, and this especially holds for policies and ethical challenges in transformed health ecosystems (41).

The broader ecosystem, with heterogeneous stakeholders, systems, services, technologies, and actors, generates ethical challenges that have to be considered as a set of “filters” to be applied to the data flow between the main actors and the pHealth digital ecosystem. Each activity and its data flow should be validated intrinsically when it is being set up. The new, pHealth-specific, ethical responsibilities are to ensure the appropriateness of each interconnection including relevance, necessity, consent, accuracy, and the ethical integrity of the whole system from the patient’s viewpoint including what is shared and what is identifiable. To better understand this, the contexts in which the pHealth ecosystem acts need to be defined, and these are determined by the technical structure and the delivery processes it supports, as shown in Figure 3.

From the technical viewpoint, the core focus of ethics is ensuring quality in building pHealth digital systems and the necessity and controls of each component and interconnection. Given the complexity of the scenario (Figure 3), a pHealth digital system should consider several types of quality principles, including at least medical device quality (as defined, for instance, in the European Medical Device Regulation 2017/745 - EU MDR) (42), software quality [as defined, for instance, in the norms “Software as a Medical Device (SaMD): Key Definitions” (43) and “Software as a Medical Device: Possible Framework for Risk Categorization and Corresponding Considerations”] (44), and data protection [General Data Protection Regulation EU 2016/679 (45) and Cybersecurity in Medical Devices: Quality System Considerations and Content FDA draft guidance 2022] (46).

As summarized in SaMD Possible Risk Categorization (44), after a system quality process is in place, it is reasonable to expect that:

- The system will perform its intended functions to meet its intended use, implying that the system responds to its requirements
- The system will be safe, so it will not create injury or damage to the users
- The system will provide a reasonable level of availability, reliability, and correct operation
- The system will be protected from cybersecurity intrusion and misuse and will ensure data protection.

### 3.2. Human context considerations

“The pHealth system covers the organization of people, institutions, and resources that deliver pHealth services meeting the health needs of individuals” (47). By definition, this takes pHealth into further ethical challenges, including the use and meaning of data on activities and relationships in addition to contextualizing personal biophysical and mental status data, as is considered below.

In the view of the WHO, ecosystem services are crucial for human well-being and health as they play an important role in the provision of basic services. “Changes in their flow affect livelihoods, income, local migration, and, on occasion, political conflict. The resultant impacts on economic and physical security, freedom, choice, and social relations have wide-ranging impacts on well-being and health” (48). Health initiatives should consider health determinants from outside and within the health system, meaning that contextual elements need to be considered when creating policies aiming to improve health (49). A key theme throughout this paper is the focus on the person, and populations, as the purpose for pHealth services but also as variable determinants of how services should be shaped, and thus meet ethical expectations.

## 4. The philosophy of personalization and the related ethical principles

### 4.1. Description of approaches to ethics

Ethics provide standards that underline our choices. It is concerned with “morality, deciding upon the right action and making the right choices in situations which arise” (50). In the view of the World Health Organization “health ethics promote the consideration of values in the prioritization and justification of actions by health professionals, researchers, and policymakers that may impact the health and well-being of patients, families, and communities” (49). Its interdisciplinary scope includes a wide range of domains which include public health, health research, and clinical care (51).

**Medical ethics** refers to the interaction between the health practitioner and the patient in the scope of clinical care, and the most widely known approach as articulated by Beauchamp and Childress has four pillars - autonomy, no maleficence, beneficence, and justice (52, 53).

**Public health ethics**, by contrast, “apply to interactions between an agency or institution and a community or population” (53) and places in the center the principles such as population health maximization, interdependence, community trust, solidarity and reciprocity, autonomy, protection of the vulnerable, and justice (54–56).

**Ethics of health research and innovation** should be based on respect for persons, concern for individual well-being, and justice across the population (57). Espousing Responsible Research and Innovation (RRI) principles research in this field should address societal needs and challenges, engage a range of stakeholders to enable mutual learning, anticipate potential problems and assess alternatives, and provide guidance on ways to proceed (58).

“Health ethic frameworks provide for a systematic analysis and resolution of conflicts through the evidence-based application of general ethical principles, such as respect for personal autonomy, beneficence, justice, utility, and solidarity” (51). Such frameworks provide fundamental methods for ethical decision-making. At the simplest level, one can identify three potential theoretical frameworks which are based on different traditions of normative ethical theories:

The **consequentialist** framework represents a pragmatic approach; this places in the center potential directions of actions and considers those who will be directly and indirectly affected. The goal is to produce the most good.

A forward-looking ethical theory originating in the work of the 19th-century philosopher Jeremy Bentham, where moral actions are judged in terms of their consequences, and a good outcome is seen as one which promotes good and avoids harm most effectively for the greatest number (59).

The **Duty** framework reflects community rules and expectations; it centers its attention on the duties and obligations with the aim of performing the correct action.

A duty or deontological approach looks backward and bases moral evaluations on the extent to which an action conforms to duties and obligations. The moral agent is required to act in good conscience sometimes irrespective of the potential for bad consequences. This approach is epitomized by Immanuel Kant (60).

The **virtue** framework based on the identification of character traits defines ethical behavior as whatever a virtuous person would do in the situation to seek to develop similar virtues (61).

An approach where in a modern context the classical list of virtues is complemented by more contemporary interpretations including the possibility of specific professional virtues.

Ethical reflections should also govern the design, development, implementation, and use of health technology innovations. Recently, Vandemeulebroucke et al. (62) systematically identified several ethical approaches to Health Technology Innovation. Their inclusion here is not to say they are to be endorsed or promoted but rather to acknowledge their influence within the disciplines of bioethics and medical ethics.

The four **Principles of Biomedical Ethics**. As mentioned earlier, as formulated by Beauchamp and Childress (52), these comprise:

- “*Respect for autonomy*” which means supporting autonomous decisions but also in the choice of whether or not to use health technology innovations and share personal information (confidentiality),
- “*Beneficence*” relies on the fact that actions are good for others because they are good in themselves,
- “*Non-maleficence*” means avoiding harmful initiatives, and
- “*Justice*” is the guardian of fairness and equality.

A **Deliberative Democratic** approach puts at the center interaction, deliberation, and basic democratic principles. It is composed of three elements:

- *Wide Reflective Equilibrium (WRE)* whose main goal is “to produce insight into the moral principles and viewpoints that stakeholders use to make their moral judgment about Health Technology Innovations” (62).
- *Accountability for Reasonableness (A4R)* identifies four conditions that guarantee that WRE processes are deliberative democratic
  ○ publicity as a guardian of transparency,
  ○ relevance as the indicator of appropriateness and acceptability for the potential stakeholders to use Health Technology Innovations,
  ○ revisability which ensures that there are methods for improvement and corrections of the processes based on newly emerging evidence, and
  ○ enforcement which ensures that “all of the above criteria must be met during a WRE process” (62).
- *Interactive Technology Assessment (ITA)* iterating emergent results with the views of stakeholders to accommodate moral, ethical, or societal issues.

Religiously Inspired frameworks are the third group of ethical approaches identified by Vandemeulebroucke et al. (62), of which two specific ones are:

- *Personalist* approaches state that humans should be considered holistically as the reference value for ethical decisions, including those considered in Health Technology Innovations. This is expressed by four principles:
  ○ Defense of human physical life is characterized by the constant respect for human life at all levels of its existence
  ○ Safeguarding the therapeutic principle means ethical acceptance when all particular conditions are met
  ○ Freedom and responsibility refer to freedom of use and responsible use of HTI
  ○ Sociality and subsidiarity are based on mutual respect among users of HIT and societal “support to those who cannot meet their own needs without undermining the place of citizens’ initiatives.”
- *Islamic* approaches consist of five principles found in sacred sources such as the Quran, Sunnah of the Prophet, Ijtihad, and the Shariah:
  ○ Protection of faith
  ○ Protection of life
  ○ Protection of intellect
  ○ Protection of progeny
  ○ Protection of property

The eponymous **AREA** framework consists of four dimensions:

- Anticipate – a constant awareness of potential difficulties with the usage and application of HIT as well as preparedness to solve the potential problems with appropriate tools or strategies
- Reflect – the identification of challenges that arise from the usage of HIT in order to “identify in advance the motivations behind the products they develop or use and to identify the results they want to achieve.”
- Engage – the involvement of all possible stakeholders whose actions are correlated with the HIT (and thus includes coproduction).
- Act – the active incorporation of the insights developed during the steps of Anticipation, Reflection, and Engagement.

The **Capabilities** approach puts human capabilities at the center. This approach developed by Nussbaum focuses on social justice and aims to show what it means for people to live a dignified life within a fair and just society (63). Dignity is considered within the context of everyday life at the family level, organization level, societal level, and national and global levels, with 10 components that can be summarized:

1. Life – being able to live meaningfully to the end of life.
2. Bodily health.
3. Bodily integrity (including freedom from assault or violence).
4. Senses – including imagination, thought, reason, and freedom of religious expression.
5. Emotions – to enable attachments to things and people.
6. Practical reason.
7. Affiliation – with and toward others and the social basis of self-respect.
8. Other species - concern and cohabitation for and with the world of nature.
9. Play - being able to laugh, play, and enjoy recreational activities.
10. Control over political and material environments.

These capabilities have been further considered and analyzed by several commentators with regard to assistive health technologies (64–66).

**Care ethical approaches** rely on the Care-Centered Framework which consists of five elements that might be used prospectively and retrospectively. There are five key elements that play a crucial role:

- The Context within which the innovation is used,
- Type of intervention for which the innovation was designed (e.g., treatment),
- Stakeholders who are playing the most significant role in the process of health policymaking,
- Type of health technology innovation which will be used, and
- Moral attitudes that are present in health policymaking.

**Casuistic** approaches claim that technological innovations in health can be assessed based on the context within which they will operate. They reject the concept of the universality of ethical frameworks. “How certain ethical principles and values were implemented in previous cases and contexts can at most indicate a certain direction for the evaluation of new HTIs” (62).

**Eclectic** approaches are combinations of the above-mentioned frameworks. Among them, there are two groups. The first one relies on ethical concepts extracted from various ethical theories; the second group draws from sociology and is mixed with ethical, bioethical, and philosophical elements.

This rich range of approaches gives the developer or policymaker the opportunity and the challenge of ensuring a balanced, open, and reasonable way forward in ensuring that pHealth developments have considered their ethical framework in a way relevant to their societal, healthcare, and infrastructure contexts. This is broadly analogous to the consideration of technical options as well where some contextual factors are already set, and the need is to design in the most effective and constructive way. Justification of optimum gains, proactively analyzing to ensure the avoidance of unintended adverse effects, and creating a positive outcome without collateral adverse effects is the prime duty of the policymakers and developers.

### 4.2. Practical assessment of ethics in pHealth

Ethics and ethical considerations branch from the identification of all the care delivery objectives and technical and contextual characteristics of the system. The health and well-being of the individual are the central focus of pHealth, with societal factors and optimum use of overall health system capabilities as related goals. As indicated throughout this paper, there is an ethical duty on developers, policymakers determining investment priorities and system characteristics, and operational staff implementing systems and service delivery to consider in conjunction with professional and citizen users the ethical issues involved and achieve a balanced prioritization of principles in a defined and defensible way. These stakeholders should engage in a democratic process of conversation, exploration, explanation, adjustment, flexibility, and understanding of different points of view to clarify why and how they have come to their positions, which should enable a positive and unambiguous objective route forward, yet with sensitivity and flexibility to facilitate necessary later adjustments.

The assessment of the ethical implications of an ecosystem depends on using a reflective and informed balance of approaches (e.g., principles of biomedical ethics, deliberative democratic, religiously inspired, etc.); the selection of the most appropriate ones will largely depend on the identified contextual factors. However, both technical and contextual factors have to be taken into account. The approach based on the widely known “principles of biomedical ethics,” if properly combined with the identified technological quality characteristics and the contextual factors, may be able to cover most of the views.

For example, the “respect of autonomy” is not ensured solely by the fact that the system works as intended, but it is crucial to consider other questions, including trust and usability – which are inter-related, and whether confidentiality and controlled sharing of key data meet the patient’s wishes. Hence, the system might need to record the acceptance by the patient as to the care options underpinning pHealth actions, who are involved including informal carers, and who can see what information and who can contribute findings, as has been considered (67–71).

Another example relates to “non-maleficence”: once a system is tested against any residual risk of harm to the patient and also in terms of cybersecurity and data protection, then it might be assumed that the system ensures the “non-maleficence” principle. However, other harms can arise if the perfectly functioning system yields the expected practical benefits to the patient, yet at the same time the patient now feels uncomfortable because the pathology now becomes evident – in effect, the patient feels stigmatized at the same time as being well-served practically.

## 5. Domains and contexts of personalized digital health

As pointed out by Blobel, “The pHealth system covers the organization of people, institutions, and resources that deliver pHealth services meeting the health needs of individuals” (47), while “The pHealth ecosystem describes the aforementioned system and the environment it interrelates with” (47). The WHO approach confirms the importance of health determinants from outside and within the health system (51). Understanding context is crucial to providing high-quality health services as it “reflects a set of characteristics and circumstances that consist of active and unique factors, within which the implementation is embedded” (49, 72), meaning that contextual elements need to be considered when creating and running pHealth systems and enabling policies, therefore recognition of contextual determinants is required while implementing Health Innovation Technologies to achieve pHealth. Contextual factors might be considered through the socio-cultural, structural (internal and external), international (extended to global), and situational factors as Leichter (73) proposed and Zdunek et al (49) modified. Contextual factors are also the starting point for key actions in the process of adaptation of digital solutions in health (Figure 2).

Socio-cultural factors relating to pHealth need to consider a wide array of issues. On the one side, societal attitudes toward digitalization have to be taken into account. These are influenced by various factors in historical and traditional views on health and well-being as well as healthcare. This is also related to tolerance and acceptance of newly emerging technologies and innovations in health in its broadest sense. The factor which will influence high or low levels of those elements is awareness. Knowledge about advantages and disadvantages related to pHealth might influence the development of an environment supportive of digital health. What is relevant here might include religion and its normative role in setting ethical and moral rules which define what is good and what is bad. The responsiveness to norms and values, which are grounded not only in religion but also in history and tradition, is expressed by the feasibility to adopt concepts of pHealth. Trust in new concepts, for instance, science, evidence, digital solutions, and policymakers will facilitate adaptation processes that enable digitalization by creating digital-friendly trends and fashions reflected in pro-digitalized lifestyles as a consequence of freedom of reasonable choice. E-literacy (33), trust in the safeguards within a proposed system, and belief in achievable benefits, as they apply at population, professional, and patient levels, will be strong factors.

*Socio-cultural determinants* in terms of pHealth include a wide array of factors, and societal attitudes towards digitalization have to be taken into account. They are influenced by

- History and traditional views on health issues
- Tolerance for and awareness of newly emerging technologies and innovation in health
- Religious aspects in terms of moral rules – defining what is good and what is bad
- Lifestyle – trends and fashions
- Level of freedom and independence, as well as the definition of freedom in the context of living
- Trust in science, evidence, policymakers, and digital solutions in health
- Family – relationships between family members, multigenerationality, and family carer involvement in the process of digitalized care/medicine
- Culture – norms, values, and symbols within the context of living
- Adaptation to change
- Fluent communication strategies
- Law
- Political ideology

*Structural determinants (external)*

- Political engagement and prioritization of digital health
- Policy preparedness for the introduction of newly emerging technologies and solutions
- The economic condition of the state and financial matters – reimbursement issues

*Structural determinants (internal)*

- Access to new technologies within the healthcare system
- Provision of health services and appropriate health infrastructure
- Skilled health workforce who will be able to design the process of implementation of new technologies
- Overall condition of the health care system and its preparedness for digitalization

*International/global determinants of pHealth*

- Connectivity
- Participation in the global institutional structures which are prompting new solutions in the scope of digital health and health innovations
- Culture of the use of evidence-based solutions emerging in other contexts
- Global evidence flow – exchange of information between agencies, structures, organizations, institutions, researchers, practitioners, and users
- Global long-standing processes such as environmental changes and climate change, which affect the paradigmatic shifts

*Situational determinants of pHealth* – unexpected events which can have their origin in:

- Global situational aspects – pandemic, war, etc.
- Behavioral situational aspects – failures of the digitalized health solutions which may discourage populations to use the innovation
- Procedural situational aspects – (un)favorable law
- Institutional situational aspects – initiatives performed at an institutional level, e.g., by regional organizations which might be facilitating or weakening the attitudes toward digital solutions.

These contextual factors may influence the values and approaches to ethical criteria, as analyzed in Section 4. In some situations, approaches may need to be modified to account for local belief systems, values, health system values, or technical infrastructures and priorities. In other cases, the strength of robustly designed digitization and personalization approaches may modify and improve some existing context restrictions.

Based on the recognition of the contextual factors, the trust level should be measured and assessed. Where it is observed that there is a high level of insecurity with the pHealth solutions, appropriate policies should be developed. The next stage is to access the population’s ability and readiness for the introduction of pHealth mechanisms. At all stages, appropriate ethical frameworks should be taken into account. The frameworks should be chosen based on the importance of contextual determinants. Where religion, history, and tradition are of high importance, the religious-based frameworks might be of importance.

## 6. Ethics, evidence, monitoring, and evaluation

### 6.1. Making ethical service implementation decisions

To be ethical, pHealth systems must be grounded on empirical objective evidence since otherwise they would be aspirational or speculative as the patient care and the business investments would be unproven. The ethical imperative for evidence-based health informatics systems and decision-making has been clearly expressed (74, 75).

Unfortunately, the availability of objective evidence is less straightforward than it might be. A lot of information may come from vendors’ or suppliers’ promises rather than from independent evidence of proof in use. Secondly, pHealth is seldom a single system but rather a build-up of components to match local care delivery circumstances and informatics infrastructures. There is also frequently an aversion by policymakers and vendors to seeing their investments subjected to searching evaluation in case it leads to suggestions of sub-optimal products or poor policymaking (76).

The soundest decisions are made when each aspect is decided based on relevant objective evidence. However, pHealth is a progressive and fast-developing scientific and service domain, and therefore waiting for solely prior in-use evidence would create stasis. It is therefore essential to ensure ethical means are found for ensuring implementations are safe and ethical, while also enabling and encouraging carefully considered innovation and improvement.

Innovation in each of these in a service investment is not about research or experiment, where the outcomes are hypothesized but not proven, and for which research protocols, ethical approval, and participant consent are required. Rather, innovation is about delivering a service in a new and more modern way compared with systems based on already existing evidence. With such innovation and context changes come risks, which should be identified objectively and then managed ethically (77, 78).

In any national or local setting, there are likely to be new elements, which will cause ‘known unknowns’ within an overall grounded service development. These may be either new component technology, or they may be pHealth functioning seen to be effective elsewhere and which there is a valid desire to emulate. Without these innovative drives, no further progress would be made, yet these innovations create new assumptions and risks.

To seek to address this conundrum, there are policies and frameworks which can be applied where a form of acceptable, controlled, and grounded speculation is needed to justify innovation as being ethically sound even though it is beyond the foundation of past performance evidence. Notable among these is the Precautionary Principle, which has been espoused by the European Commission as a policy touchstone and provides safeguarding of the population by ensuring new risk is considered and appropriately mitigated; three other frameworks enable anticipatory objective formulations of expectations.

The **Precautionary Principle** of not letting new risks run unchecked has been very helpfully codified by the European Commission to apply to science-based innovation (79). Though sometimes erroneously portrayed as hampering innovation, this policy is intended to enable scientific progress without letting the population be exposed to unquantified risk. It requires risks of an as yet unvalidated innovation to be assessed and mitigated through controls based on six rules:

Controls should be

- proportional to the chosen level of protection
- non-discriminatory in their application
- consistent with similar measures already taken
- based on an examination of the potential benefits and costs of action or lack of action (including, where appropriate and feasible, an economic cost/benefit analysis)
- subject to review, in the light of new scientific data
- capable of assigning responsibility for producing more scientific evidence

Noteworthy is that this is part of a hierarchy of deepening evidence gathering, and the possible risk from the innovation must be weighed up alongside the cost of inaction on that innovation.

Then, of the three anticipatory methods:

**Transferability** relates to whether something which works in one health system or population setting will work identically in another. The components are not new, but the hypothesis that they will work in the same way in a different operational and service setting cannot be assumed, and indeed major challenges and risks may occur, ranging from data feeds to societal acceptance. The Population, Intervention, Environment, and Transfer (PIET) model shows how to assess these four domains (80).

**Update Equivalence** is whether updated components or different inter-relationships of components will operate as planned based on previous versions. This is a significant conundrum in many fields including pharmaceuticals, medical devices, and even aviation (81). Both the new material or component and its interaction both with its embedded system and with users must be objectively considered, planned benefits scrutinized, and possible unanticipated effects considered and either ruled out or guarded against.

**Evidence Synthesis** is the technique whereby evidence obtained in one setting is reviewed to identify the dependence on context and the influence of specific aspects, so as to enable a reasoned hypothesis of potential performance and outcomes in a new setting or with a new component (82).

### 6.2. Monitoring

No system, technology, or skilled team is likely to run exactly to expectations when first instigated. This makes it important to put in place monitoring arrangements, which may be close and frequent examination in the early days to identify any teething problems or failures to meet specifications or acceptance. Even when a system has run according to plan from the outset, it is important to continue regular monitoring as equipment or its use can deteriorate, staff start to work less rigorously, or new staff may not be trained to the initial standard. However, monitoring is not just about finding problems though this is important; monitoring may also find unexpected improvements, for instance, as staff becomes more proficient and users increasingly accept the innovations or other benefits such as better service outcomes.

Monitoring should relate to the three stages identified in Figure 2, namely, objectives, design, and use, while the area to be monitored should match the technology and interest areas of Figure 3. The metrics used should relate to the business plan and clinical protocols which should be at the core of any pHealth or other clinical system.

The Donabedian triptych of Structure, Process, and Outcome forms a useful framework (83, 84). The structure includes technical equipment, infrastructure, and allocated staff establishment, and monitoring will show if the intended pattern and levels continue to be present. The process will include the patient flow and key points of pHealth interactions, but it should also include equipment availability and response times, as well as whether responses were compromised by additional use or competing traffic. The outcome should include costs and numbers treated compared with the business plan, clinical outcomes against expectations, and user acceptance and satisfaction. Again, target values should have been set in the business and implementation plans.

### 6.3. Evaluation

While monitoring is focused on how the implementation is working in real life, Evaluation is a more holistic appraisal, often run by an expert third party (74, 75, 85). Evaluation requires systematically examining each aspect of the design and running of a system, and the International Medical Informatics Association has endorsed both a methodology (86) and a standard for reporting such studies (87), which itself has been accepted by the Enhancing the QUAlity and Transparency Of health Research (EQUATOR) system (88). Conducting evaluation studies is important to underpin the claims of the technological support sector and provide the body of evidence needed for informed policymaking. Analyses have been carried out on the volume and focus of past evaluation studies (89, 90) and on the need for a systematic forward view (91). pHealth, as a newer and key informatics application area bringing personalization to patients through digital processes, needs to demonstrate its commitment to expanding its evidence base by means of evaluation studies so as to progress in this important discipline (92).

### 6.4. No blame reporting

Another key aspect of an ethical implementation is that any person using the system should be able to report any problem or apparent fault or error that they perceive. The obvious objective is to ensure that risks or faults are corrected and is similar in principle to reporting other areas of health care such as medication errors. While IT systems can indeed have faults that need reporting, which can include failure to display past results when relevant, correctly functioning informatics applications including pHealth can have aspects that users do not fully understand – either because they are not intuitive to users (clinical or lay) or because staff have not been adequately trained or do not have access to relevant documentation. This is particularly important with the inclusion of AI given its invisibility of origin and process. Thus, no-blame reporting is an effective means of identifying and rectifying these softer areas aspects of user-perceived or user-believed problems as well as technical problems, ensuring that systems are trusted and therefore optimally used.

## 7. Conclusion

Personalized digitally supported health is the ideal of health care philosophy and purpose but has ethical challenges along the way caused by the concepts, novelty, and need for a wide range of new care delivery approaches to achieve effective personalization, while at the same time using varied and innovative technologies. The road to pHealth is paved with good intentions, but there are pitfalls on the route.

By its nature, pHealth has the potential to create suspicion and negativity if perceptions of black-box thinking, system determination of treatments and actions, and domination by impersonal technology are allowed to take hold. Therefore, to achieve their goal, pHealth system creators must engage with ethics at all stages of design, build, and operation in a number of ways – adherence to established ethical principles, openness and inclusivity with stakeholders, and concurrent review and evaluation; moreover, these must be related to societal and system contexts.

Taking a proactive open approach to ethical commitment and methodology is not only morally right but will be rewarded with an aura of trust and of person-centered philosophy which will strengthen the general appeal of pHealth.

The 5P medicine methodology and the related ISO 23903 standard are designed to address this. This paper has sought not only to argue the case but also to outline the range of methods available to be selected and used according to the service area, system type, and context of service delivery.

## Data availability statement

The original contributions presented in the study are included in the article, further inquiries can be directed to the corresponding author.

## Author contributions

MR assembled the author team, facilitated virtual panel discussions, and acted as facilitator and integrating editor. All authors contributed material from their own dimension and expertise, equally responded to ongoing revisions, and agreed the final manuscript.

## Funding

Publication costs of this study were funded from the legacy bequeathed by the late Prof dr ir J. C. Wortmann of Groningen University to promote ethics studies in health.

## Conflict of interest

The authors declare that the research was conducted in the absence of any commercial or financial relationships that could be construed as a potential conflict of interest.

## Publisher’s note

All claims expressed in this article are solely those of the authors and do not necessarily represent those of their affiliated organizations, or those of the publisher, the editors and the reviewers. Any product that may be evaluated in this article, or claim that may be made by its manufacturer, is not guaranteed or endorsed by the publisher.
